# Computed tomography accelerates staging in patients with Merkel cell carcinoma

**DOI:** 10.1007/s00405-018-5150-x

**Published:** 2018-09-28

**Authors:** Elisabeth Foki, Alexandra Fochtmann-Frana, Georg Haymerle, Stefan Nemec, Benjamin Loader, Christos Perisanidis, Boban M. Erovic

**Affiliations:** 1 Department of Otorhinolaryngology, Head and Neck Surgery, Medical University of Vienna, Währinger Gürtel 18-20, 1090 Vienna, Austria; 2 Department of Surgery, Clinical Division of Plastic and Reconstructive Surgery, Medical University of Vienna, Vienna, Austria; 3 Department of Radiology, Division of Neuroradiology and Musculoskeletal Radiology, Medical University of Vienna, Vienna, Austria; 4 Department of Otorhinolaryngology, Head and Neck Surgery, Rudolfstiftung Teaching Hospital, Vienna, Austria; 5 Department of Oral and Maxillofacial Surgery, Medical University of Vienna, Vienna, Austria

**Keywords:** Imaging, Diagnosis, Cancer, Skin, Ultrasound, Computerized tomography

## Abstract

**Purpose:**

No imaging algorithms for diagnostic imaging in patients suffering from Merkel cell carcinoma (MCC) have been established so far and thus staging work-up is challenging. Long presentation-to-treatment intervals determine further treatment course and, consequently, have an impact on clinical outcome in patients with MCC.

**Methods:**

In this retrospective study, diagnostic imaging of 37 MCC patients was analyzed. CT, ultrasound, and PET/PET–CT imaging for primary staging work-up with time frames from patients´ initial presentation and imaging until completion of tumor staging were analyzed.

**Results:**

Tumor staging could be completed earlier when (1) less examinations (35 vs. 42 days) were carried out or (2) computed tomography was used as the initial imaging modality (28 vs. 35 days). Furthermore, CT imaging, when used as the initial imaging study, was linked to less follow-up imaging (3 vs. 6).

**Conclusion:**

Computed tomography as the first-staging imaging technique in MCC patients leads to less follow-up studies and fastest completion of tumor staging.

## Introduction

Merkel cell carcinoma (MCC) is a rare and highly aggressive neuroendocrine tumor of the skin with a significantly higher mortality rate than melanomas [1]. The previous results showed an age-adapted threefold increase of MCC incidence between 1986 and 2001 [2]. Thus, diagnostic work-up, histopathologic verification, and disease management of patients with Merkel cell carcinoma is becoming more important.

Due to its similarity to benign skin tumors, the diagnosis of Merkel cell carcinoma can be challenging [3]. Patients typically present with new, rapidly developing skin lesions. MCC appears characteristically as firm, non-tender, dome-shaped, red, purple, or violet nodule [4, 5]. The overlying skin is smooth and shiny, sometimes exhibiting ulcerative, acneiform, or telangiectatic signs [4, 6]. It affects mainly the sun-exposed area of the head and neck [7, 8], each at a rate of 40% [9]. Clinically MCC spreads rapidly to regional lymph nodes and in 50% distant metastasis occur, predominantly in the liver, bone, brain, and the lungs [6, 7]. The 5-year survival rate for patients suffering from a primary tumor without metastasis is 75%, whereas it is 59% for patients suffering from local recurrence and/or lymph-node metastasis [9]. The 2-year disease-specific survival rate is dismal, with an 11% estimation, when distant metastatic disease is detected [9]. Due to the aggressive behavior of this tumor, rapid diagnosis and staging work-up are essential for treatment, since prolonged waiting times are linked to worse clinical outcome in patients with cancer [10–13].

However, still, to date, there is sparse literature on imaging algorithms or widely accepted guidelines for imaging in patients with Merkel cell carcinoma [3, 4]. Recently published literature looked in particular at the diagnostic impact of various imaging methods such as computed tomography, sonography of the lymph nodes, and PET/PET–CT [3, 4, 7].

Hawryluk and colleagues recommended 18-FDG PET for the detection of bone metastasis [14]. However, it has limited value for detection of liver metastasis. In the detection of lymph-node involvement, PET–CT has a sensitivity and specificity of 83% and 95%, respectively [14]. In a study of Concannon, it altered staging in 33% of all cases as it was more sensitive than clinical examination, CT, as well as MRI [15].

Peloschek and colleagues recommended ultrasonography as the initial imaging method in staging of patients with Merkel cell carcinoma. In easy accessible lymph-node regions, ultrasonography is cost-effective and highly accurate [7]. In the same study, the authors could show that computed tomography has a specificity of 96.2% and a sensitivity of 89.1% for imaging of lymph-node involvement and distant metastasis in MCC patients [7]. A meta-analysis of Liao showed that CT and ultrasonography have a similar sensitivity, but CT is superior in regards to specificity in lymph-node mapping of the head and neck area compared to the ultrasound [16].

Summarizing the literature of imaging of MCC, the major diagnostic tools for staging are CT, lymph-node sonography, and PET or PET–CT. The objective of this study was, hence, to analyze whether one of the aforementioned diagnostic methods or the overall number of imaging techniques performed influences time and accuracy of MCC staging.

## Materials and methods

### Patients

Retrospective data of 37 patients were available (20 females and 17 males). Mean and median age at the time of first diagnosis of Merkel cell carcinoma was 72.5 and 76 years, respectively (range 46–87 years). All patients were treated at the Departments of Dermatology and Otorhinolaryngology, Head and Neck Surgery, at the Medical University of Vienna. Only patients diagnosed with a new and untreated Merkel cell carcinoma between 1994 and 2012 were included in this study. Demographic, clinical, and pathological data were obtained from hospital records. Approval of the institutional research ethics board was retrieved before performing this study (REB# 1798/2013).

### Statistics

Demographic and pathologic data were summarized using descriptive statistics. Outcome measures were number of radiologic examinations and time interval for imaging and completion of tumor staging. In particular, the time frame between primary presentation and first imaging (FI) and the total time interval between primary presentation and completion of radiologic staging before treatment onset (CS) were determined (days). Imaging studies were counted as baseline examinations if the examination was performed promptly to the first physicians’ visit either in an outpatient setting or in a hospital and if it was realized before the onset of treatment. An imaging study was defined as imaging of a predefined part of the body, e.g., the cervical lymph nodes or computed tomography of the abdomen. As a consequence, we counted three examinations when (1) computed tomography of the head and neck, (2) thorax, and (3) abdomen was done in one patient until staging was completed.

During the staging process, the majority of our patients were seen more than once, and subsequently, imaging studies were performed until the final staging was completed. Histologic data were not included in our data evaluation.

Demographic and pathologic data were summarized using descriptive statistics. Mean, median, and standard deviation were calculated. False-negative and false-positive results of the conducted examinations were evaluated in concordance with the available reports. Examinations were stratified according to their accuracy and it was taken into concern if staging was altered during the course of treatment. However, if staging was altered after 6 months after the first diagnosis, it was counted as recurrent disease. Sensitivity and specificity of the imaging methods were calculated. Cost-effectiveness of conducted examinations was calculated according to the routine costs in our environment using one-way analysis of variance and *t* test, respectively.

### Staging

Seventeen, fourteen, and two patients with stage I (46%), stage II (37.8%), and stage III (5.4%) diseases were included in this study, respectively. According to the seventh edition of the AJCC cancer system, stage I is defined as tumor size ≤ 2 cm across, stage II is defined as tumor size > 2 cm across, and stage III is defined as any size of primary tumor invading nearby lymph nodes. In 4 (10.8%) of 37 patients, no primary tumor was found. Those patients were defined as patients with cancer of unknown primary. In 51.4% (*n* = 19), the primary tumor site was within the head and neck areas, in 27% (*n* = 10) on the upper and lower limbs, and in 21.6% (*n* = 8) the trunk. Four (10.8%) patients suffered from recurrent disease.

Surgery was performed in 46% (*n* = 17), primary radiation treatment in 5.4% (*n* = 2), surgery and subsequent radiation treatment in 40.5% (*n* = 15), adjuvant chemoradiation therapy in 5.4% (*n* = 2), and surgery followed by chemotherapy in 2.7% (*n* = 1) patients. Twenty-eight patients were treated with curative intention (75.7%) and nine patients were treated with palliative intention (24.3%). Twenty-four patients (64.9%) had to undergo re-resection to achieve negative resection margins.

### Imaging

Thirty-seven patients underwent a total of 168 radiologic examinations before staging was confirmed. The mean number of investigations per patient was 4.5 (range 2–8). In 78.4% of all patients, one or more examinations with computed tomography of the neurocranium, thorax, or abdomen were performed (*n* = 29). Contrast agent-containing iodine was used routinely. Sonography of the cervical, axillary, and inguinal lymph nodes was performed in 62.2% of the patients (*n* = 23), and seven patients underwent a PET or PET–CT scan (19%). Further imaging methods such as octreotide scintigraphy, MRI, or X-ray of the bones and lungs were sub-summarized as “various examinations” as they were performed in less than three patients.

## Results

### Waiting times for all MCC patients

The median time until the first imaging method was performed, and staging was completed was 19 and 35 days, respectively. The mean value in days for FI and CS was 25 (SD: 23 days), and 38 (SD: 21 days), respectively.

## Group stratification according to the count of examinations

**Fig. 1:**
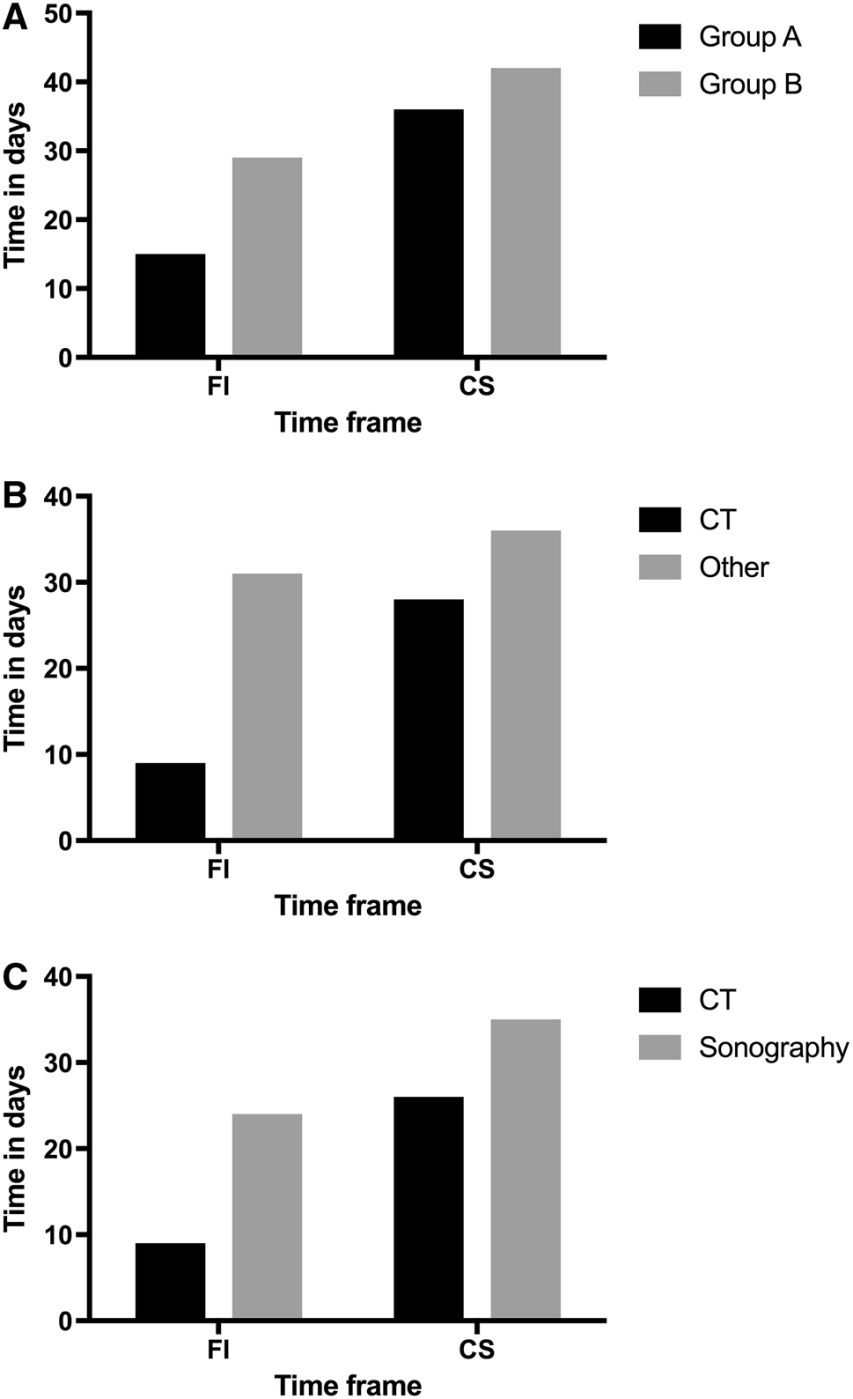
Comparative data of patients according to number of imaging methods and imaging methods. **a** Patients undergoing a lower number of imaging methods (Group A) showed a shorter FI (15 vs. 29 days) and CS (35 vs. 42 days), respectively, compared to group B. **b** Performing a CT scan at the initial presentation of the patient led to a shorter FI (9 vs. 31 days) and CS (28 vs. 36 days) compared to other patients. **c** However, lymph-node sonography at the initial presentation but not computed tomography led to a longer FI (24 vs. 9 days) and CS (28 vs. 35 days). *FI* time interval from first consultation to first imaging, *CS* time interval to completion of tumor staging

Patients who underwent four examinations or less (*n* = 19, 51.4%) or less until tumor staging was completed were stratified into group A and patients with five examinations or more (*n* = 18, 48.6%) were stratified into group B (Fig. 1; Tables 1, 2).

**Table 1:** Demographic data of 37 patients with Merkel cell carcinoma

Patient	No.^a^	Group	FI^b^	CS^c^	Stage	Rec.^d^	^e^
1	3	A	20	36	1	0	ANED
2	4	A	48	54	1	0	DNED
3	7	B	36	54	2	0	DOD
4	8	B	89	89	1	0	ANED
5	7	B	19	28	1	0	DNED
6	3	A	33	47	1	0	DNED
7	7	B	40	40	1	0	DNED
8	2	A	15	16	1	0	DOD
9	5	B	82	82	1	1	DOD
10	5	B	0	35	2	0	DOD
11	5	B	10	51	2	0	DNED
12	2	A	19	19	4	0	ANED
13	4	A	49	49	1	0	DNED
14	4	A	65	70	2	0	ANED
15	4	A	35	35	1	0	DNED
16	5	B	47	75	1	0	DNED
17	5	B	3	10	4	0	DOD
18	2	A	22	39	2	1	DOD
19	3	A	2	33	1	0	ANED
20	4	A	6	57	2	0	DOD
21	3	A	41	41	3	0	DOD
22	5	B	5	12	2	0	DOD
23	6	B	27	21	2	0	DOD
24	4	A	3	18	4	0	DNED
25	2	A	10	50	2	0	DOD
26	6	B	33	48	1	0	DNED
27	2	A	35	35	2	0	DNED
28	8	B	16	48	3	0	DOD
29	6	B	34	35	1	0	DNED
30	7	B	31	43	1	0	DNED
31	7	B	11	29	2	1	DOD
32	2	A	6	20	1	0	DNED
33	2	A	122	122	1	0	ANED
34	2	A	0	12	2	0	DNED
35	6	B	78	78	2	0	DNED
36	3	A	11	11	4	1	DOD
37	8	B	7	29	2	0	ANED

*No*. number of examinations, *FI* time interval from first consultation to first imaging, *CS* time interval to completion of tumor staging

^a^Number of examinations

^b^Time from first consultation to first imaging

^c^Time from first consultation to completion of tumor staging

^d^Recurrent disease

^e^Status of survival: *ANED* alive with no evidence of disease, *DNED* died with no evidence of disease, *DOD* died of disease

**Table 2:** Demographic data of patients with Merkel cell carcinoma according to imaging method

Pat.	No.^a^	Group	FI^b^	CS^c^	Sono^d^	CT	Combi^e^	PET/CT
1	3	A	20	36	1	1	1	0
2	4	A	48	54	1	0	0	0
3	7	B	36	54	1	1	1	0
4	8	B	89	89	1	1	1	0
5	7	B	19	28	1	0	0	0
6	3	A	33	47	0	1	0	1
7	7	B	40	40	1	1	1	0
8	2	A	15	16	0	1	0	0
9	5	B	82	82	1	1	1	0
10	5	B	0	35	0	1	0	0
11	5	B	10	51	1	1	1	0
12	2	A	19	19	0	1	0	0
13	4	A	49	49	0	1	0	0
14	4	A	65	70	0	1	0	0
15	4	A	35	35	1	0	0	0
16	5	B	47	75	1	1	1	0
17	5	B	3	10	0	1	0	1
18	2	A	22	39	0	1	0	0
19	3	A	2	33	1	0	0	0
20	4	A	6	57	0	1	0	0
21	3	A	41	41	0	1	0	0
22	5	B	5	12	1	1	1	0
23	6	B	27	21	1	1	1	0
24	4	A	3	18	0	1	0	1
25	2	A	10	50	0	1	0	0
26	6	B	33	48	1	0	0	0
27	2	A	35	35	1	0	0	1
28	8	B	16	48	1	0	0	1
29	6	B	34	35	1	1	1	0
30	7	B	31	43	1	1	1	0
31	7	B	11	29	1	1	1	1
32	2	A	6	20	0	1	0	0
33	2	A	122	122	0	1	0	0
34	2	A	0	12	1	0	0	0
35	6	B	78	78	1	1	1	0
36	3	A	11	11	1	1	1	0
37	8	B	7	29	1	1	1	1

*No*. number of examinations, *FI* time interval from first consultation to first imaging, *CS* time interval to completion of tumor staging, *Sono* sonography, *Comb* combination of sonography and computed tomography

^a^Number of imaging methods

^b^Time from first consultation to first imaging

^c^Time from first consultation to completion of tumor staging

^d^Sonography

^e^Combination of CT and sonography

### Group A

In group A (*n* = 19, 51.4%), 5 patients underwent sonography of the lymph nodes (26.3%) and 12 patients underwent computed tomography for the initial staging work-up (63.2%). Only two patients had lymph-node sonography combined with computed tomography (10.5%). FI accounted for 15 days (median value; mean = 19, SD: 16 days), whereas CS was completed after 35 days (median value; mean = 31, SD: 17 days).

### Group B

Group B consisted of 18 patients; 3 (11.1%) patients underwent sonography of the lymph nodes and 2 had computed tomography imaging (16.7%) alone. In 13 (72.2%) patients, ultrasound and CT scanning of the lymph-node basins was performed. In this group, patients completed FI and CS after 29 (median value; mean = 32, SD: 27 days) and 42 (median value; mean = 45, SD: 24 days), respectively.

## Group stratification according to the initial imaging method for staging

### Computed tomography as the initial imaging strategy

Patients who underwent computed tomography of any body region (*n* = 14, 48.3%) during radiologic staging, had to undergo 3.2 examinations (median value; median = 3, SD: 1.2) before staging was completed. Median FI accounted for 9 days (mean = 16, SD: 16 days) and staging was completed after 28 days (median; mean = 30 days, SD: 18 days).

In contrast, in 15 patients (51.7%), the imaging process was conducted without using computed tomography. Those patients underwent 5.4 investigations (mean value; median = 6, SD: 2). FI was 31 days (median; mean = 31, SD: 25 days) and CS was completed after 36 days (mean = 43 days, SD: 22 days). (Table 3).

**Table 3:** Time frames for diagnostic imaging of patients suffering from Merkel cell carcinoma according to group and imaging method

Modality	FI^a^	CS^a^
Group A		
Median	15	35
SD	16	17
Mean	19	31
Group B		
Median	29	43
SD	27	24
Mean	32	45
CT		
Median	9	28
SD	16	18
Mean	16	30
Sonography		
Median	24	35
SD	17	13
Mean	26	37
Combination		
Median	31	40
SD	28	25
Mean	35	46
PET–CT		
Median	11	29
SD	2	14
Mean	15	31

*Group A* patients who underwent less than five examinations, *Group B* patients who underwent five or more examinations, *FI* time interval from first consultation to first imaging, *CS* time interval to completion of tumor staging

^a^Values represent time frames in days

When patients (*n* = 8, 21.6%) received sonography of one or more lymph nodes without computed tomography, the mean number of examinations was 4.5 (median = 4, SD: 2). FI was done after 24 days (median, mean = 26, SD: 17 days) and CS was completed after 35 (mean = 37 days, SD: 13 days) (Table 3).

### Combination of lymph-node sonography and computed tomography as primary imaging modality

Patients who received a combination of computed tomography and lymph-node sonography (*n* = 15) underwent a mean of 5.8 examinations per patient (median = 6, SD: 1.6). The time from first presentation to first imaging was 31 days (median value; mean = 35, SD: 28 days). CS was completed within 40 days (median value; mean = 46 days, SD: 25 days) (Table 3).

### Use of PET or PET–CT as staging strategy

Those patients (*n* = 7) who had a PET or PET–CT underwent a mean number of investigations of 5.3 (median = 5, SD: 2 days). FI and CS accounted for 11 (mean = 15, SD: 14 days) and 29 (mean = 31 days, SD: 14 days), respectively (Table 3).

## Sensitivity and specificity of conducted examinations

After re-evaluation of all imaging methods, tumor staging was changed in 13.5% of all patients (*n* = 5). In one case, computed tomography showed false-positive, and in two cases, false-negative dissemination of the primary tumor, with a sensitivity of 80% and a specificity of 95%. Similar rates could be determined for sonography with a rate of 82% for sensitivity and 100% for specificity (Table 4).

**Table 4:** Data of false-positive and false-negative results of imaging methods in Merkel cell carcinoma according to imaging modality

Modality	Disease	Staging	Re-staging^a^	By^b^
CT	Pulmonary metastasis	T1N0M1	T1N0 M0	PET–CT
	Pelvic lymph nodes	TxN0M0	TxN1M0	biopsy
	Cervical lymph nodes	T1N0M0	T1N1M0	PET–CT
Sonography	Inguinal lymph nodes	TxN0M0	TxN2M0	CT
	Inguinal lymph nodes	T1N0N0	T1N1M0	sonography

^a^Re-staging after re-evaluation by another diagnostic tool

^b^Imaging method or diagnostic tool that led to correct staging

## Cost-effectiveness of conducted examinations

Neither use of computed tomography or sonography, nor the combination of those modalities did influence cost-effectiveness significantly (*p* = 0.48). Not surprisingly, use of four examinations or less led to significantly less costs than use of more than four examinations (*p* = 0.001).

## Discussion

The key role of imaging in patients suffering from MCC is staging of the tumor by determining the size and localization of the primary lesion and identifying loco-regional invasion including lymph nodes and soft tissue [7, 17, 18]. In the current literature, sonography, computed tomography, and PET–CT are discussed as the major diagnostic tools in patients with MCC. In this study, we evaluated these three imaging methods to determine their efficacy and accessibility. To our knowledge, this is the first study to show that the choice of the imaging method influences staging success and time until treatment onset. Time delay in staging should be avoided, as it is not only cost-intensive, but also a significant contributor to postponed onset of treatment, being associated with a significantly worse outcome and decreased health-related quality of life during waiting times [2, 13].

In our study, the staging work-up was conducted with a mean number of 4.5 radiologic examinations. We clearly acknowledge that a lower number of imaging lead to shorter duration of the staging process and, subsequently, to an early initiation of therapy.

Use of computed tomography seems to accelerate the staging process, as fewer examinations were necessary for staging work-up (3 vs. 6). Computed tomography is independent of physicians’ skills and all scans can be considered by other clinicians, during rounds and in tumor boards. The majority of incorrect results in our cohort occurred in sonographic inguinal lymph-node staging. However, despite the low sample size, lymph-node staging of the cervical lymph nodes seems to be more accurate than for abdominal lymph nodes. Nevertheless, we hypothesize that the difference in CS between CT and ultrasonography indicates that sonography is very accurate in providing fast information regarding locally invasive and/or metastatic Merkel cell carcinoma disease but lacks in regards to lymph-node mapping and reproducibility. Therefore, further examinations and, in most of our patients, CT imaging were needed to complete tumor staging.

A very limited number of MCC patients in our cohort underwent PET and PET–CT imaging. Recent publications claimed the usefulness of PET–CT in staging of MCC [15, 19]. However, due to the high costs of a PET–CT scan and a limited availability compared to in particular CT and ultrasonography, it is not always useful for routine staging of carcinoma patients. In our study, PET or PET–CT did not show any benefit concerning the number of imaging examinations and, of significant importance, a time delay was observed compared to those patients who received computed tomography and ultrasonography until tumor staging was completed. Interestingly, in all patients, PET or PET–CT was never used as the sole diagnostic tool, which challenges the usefulness of this very expensive examination. However, in a few cases, scintigraphy was useful for the exact diagnosis of secondary lesions.

We analyzed cost-effectiveness of all conducted examinations according to the dominant diagnostic method. No significant difference in cost-effectiveness according to the different imaging modalities was detectable. However, this might be contributed to the low patient number included in this study. Notably, the delay in staging and treatment initiation and a consequently prolonged hospitalization period, which results in a multiplication of incurring costs, has not been taken into account in this analysis.

In general, time delay in staging should be avoided, as it is not only very cost-intensive but also a significant issue for postponed onset of treatment. As previously shown by the group of Tsang, delay of treatment initiation is associated with a significantly worse outcome [2] due to the very aggressive, rapidly growing characteristics of this carcinoma. This is consistent with the other studies showing a negative prognostic influence of waiting times on outcome in head and neck squamous cell carcinoma, breast cancer, and non-small lung cancer [10–12]. Moreover, studies showed that carcinoma patients have a significantly decreased health-related quality of life during waiting times for surgery, and this leads to impairment of vitality and mental health [13]. Thus, we propose prioritization by implementation of an elapsed-time-of-waiting system to improve access to imaging for aggressive diseases [20].

## Conclusion

Our study clearly shows that a huge panel of different imaging methods was and is still used for staging in Merkel cell carcinoma patients. This subsequently leads to a prolonged time interval between imaging and treatment onset. We could show that the use of computed tomography as the first imaging method poses an advantage for tumor staging as it accelerates staging work-up and reduces the number of further examinations. Therefore, we strongly recommend that CT should initially be used for tumor staging in patients with Merkel cell carcinoma.
